# The Spontaneous Disappearance of a Sarcomatous Tumour

**Published:** 1900-03

**Authors:** G. Munro Smith

**Affiliations:** Surgeon to the Bristol Royal Infirmary


### THE SPONTANEOUS DISAPPEARANCE OF A SARCOMATOUS TUMOUR.

BY

G. MUNRO SMITH, M.R.C.S.,

*Surgeon to the Bristol Royal Infirmary.*

---

A FEW reports of the spontaneous cure of malignant growths have from time to time appeared in various medical journals. But owing to the difficulty of making an exact diagnosis in such cases, the nature of the tumour has generally been called in question and the malignancy doubted. The following record adds to the meagre evidence we have on the subject.

W. R. C., aged 52, gardener, was admitted to the Bristol Royal Infirmary, under the care of Dr. James Swain, in March, 1894. He had, since the previous November, noticed a swelling at the angle of the jaw, on the right side, which had slowly increased in size and had caused irritation but no pain. The man was in apparently good health, and there was no history of syphilis, tubercle, or preceding lesion of the skin of the part affected.

Dr. Swain removed the growth, which was found to be encapsuled but firmly attached to the masseter and other structures, and very vascular. It dipped under the jaw and was adherent to the sub-maxillary gland. It measured about two inches by one inch in diameter. A small gland was removed with it.

On examination it was found to consist of a soft globular nucleus surrounded by an envelope of yellowish grey material; and outside this was a third zone, darker and softer.

Microscopically small round cells were the chief constituent of all three zones. There was very little stroma.

The wound healed and the man went out and resumed his work; but was re-admitted to the Infirmary, under my care (Dr. Swain being away at the time), on August 10th, 1895, with a smooth, firm, lobulated swelling involving the parts behind and beneath the angle of the jaw (right side), and extending downwards anterior to the sterno-mastoid. There was some loss of flesh and slight pain. The tumour was increasing with some rapidity. He had no other swellings.

It was considered that this was a recurrence of the original tumour, and an operation was decided on.

On August 13th, I made a long incision and exposed the superficial part of the growth. It was encapsuled and smooth, but adherent to the surrounding tissues, and extended deeply into the neck, being firmly adherent to the prevertebral muscles, &c. There was very free hemorrhage, which owing to the depth of the wound was restrained with great difficulty; and it was found to be quite impossible to remove the tumour. The wound was plugged with gauze. In the evening more bleeding occurred, and artery forceps had to be left on several bleeding points. There was no chance, therefore, of healing by first intention, but the wound slowly granulated up, and in three weeks' time was almost healed.

The patient left the Infirmary and went to stay with friends at Torquay for two or three weeks. On his return I was surprised to find a very marked diminution in the size of the tumour, and the man was putting on flesh. He went away again, to the Isle of Wight and then to Wolverhampton, returning to Bristol at the end of December, four and a half months after the operation. He then appeared perfectly well, and there was no trace whatever of the swelling. He had, however, a small hard gland on the opposite side of the neck.

In February, 1896, he again attended at the Infirmary with enlargement of his right tonsil. A circular ulcer gradually formed, with hard, everted edges, involving the anterior pillar of the fauces and tonsil. Whilst he was attending for the throat he was put upon full doses of iodide of potassium, but he rapidly emaciated, his tongue and breath became very foul, and his general appearance was markedly cachectic. He grew steadily worse; eating and drinking became difficult, and after some weeks I was not surprised to have a message from his wife that he was too ill to attend as an out-patient, and was confined to his house. Nevertheless, without any special treatment, beyond rest in bed and antiseptic washes, this malignant-looking ulcer gradually but completely healed, and the emaciation and cachexia began to improve rapidly.

In July, 1896, the patient came to the Redland Dispensary, and attended under Mr. J. Griffiths (who kindly furnished me with notes), with a round lump just beneath his *left* ear, firm, pigmented and slowly increasing in size. He also complained of swelling of the abdomen, and on examination it was found that the abdominal walls were tense from the presence of ascites. There was much œdema of the lower limbs and scrotum. There was no albumin or other abnormal constituent in the urine, no jaundice, no hepatic enlargement, and no cardiac defect could be discovered. Besides the growth under the left ear, there were two or three small subcutaneous nodules on the arms and legs. The diagnosis of malignant growth was made, and the case was considered hopeless. But in about seven weeks' time the dropsy began to disappear, and gradually left an apparently healthy abdomen.

Meanwhile the lump on the left side of the neck began to increase, and in December, 1896, he was again admitted to the Infirmary under my care. This lump was the size of a small orange, fungating, soft, and dark reddish brown in colour. It frequently bled from the foul cauliflower-like excrescences with which it was covered. As the patient was becoming very weak from loss of blood, I removed this large superficial tumour. Its base was indurated, involving the deep structures of the neck, and no attempt was made to remove this.

Some attempt at healing took place, and the man left the Infirmary improved in health. He had, however, in the groins, and on the forearms and legs, several deeply pigmented lumps, presenting all the appearances of sarcomatous deposits. Microscopical examination of the removed tumour showed small round cells, with numerous blood-vessels and very little stroma.

These subcutaneous nodules rapidly developed, and became very numerous. He died at his house on April 6th, 1897, from exhaustion. He had twenty-two of these pigmented tumours on the right leg alone. Unfortunately no *post-mortem* examination was made.

It is impossible to review this case without astonishment. The primary lesion appears to have been a lympho-sarcoma at the angle of the jaw. This was extirpated; it recurred, and after an unsuccessful attempt at removal of the recurrent growth, the latter spontaneously disappeared. The ulcer on the tonsil and palate and the ascites seemed clearly due to malignant disease, yet recovery from both took place. The microscopic examination, the clinical features, and the termination by numerous pigmented tumours &c., causing death from exhaustion—all this is strong evidence in favour of the whole cycle of events being malignant.

Could any of these lesions be fairly attributed to syphilis? There is no good reason for thinking so. Quite apart from the general clinical picture of malignancy in this case, it may be said that syphilis rarely, if ever, causes large glandular tumours. The resemblance between tertiary ulcer of the tonsil and sarcomatous disease of the part has, however, been commented on,[1] and it may be noticed that Esmarch[2] expresses a strong opinion that certain sarcomatous tumours are *caused* by syphilis. Those that are improved by arsenic and erysipelas toxins he places in this category.

The so-called lympho-sarcomata should undoubtedly be put in a separate class from the other sarcomata, both on account of their clinical features, their mode of origin, and their structure. It may be said, in fact, that any irritation in lymphatic glands causing hypertrophy may give rise to sarcoma of this nature. G. Ricker, for instance, has reported two cases showing the apparently close relationship between lympho-sarcoma and tuberculosis, demonstrating that the tubercle bacillus may provoke a growth of great malignancy; and Senn[3] lays stress on the *rôle* played by injury and irritation. Nevertheless, primary sarcoma of the lymphatics must be looked upon as comparatively rare.

Even with the microscope an exact diagnosis of these

[1] Vide *N. York M. J.*, 1897, lxvi. 769.

[2] *Ber. ü. d. Verhandl. d. deutsch. Gesellsch. f. Chir.*, 1895, xxiv. 23. [Supplement to *Centralbl. f. Chir.*, 1895, No. 27.]

[3] *The Pathology and Surgical Treatment of Tumors*, 1895.

tumours is obviously difficult, and it is therefore foolish to assert that any swelling which showed on section merely small round cells and followed a malignant course for a time—ultimately disappearing—must be a sarcoma. But the after-history of my unfortunate patient, and the combination of pathological and clinical features, lead to the conclusion that he recovered for a time from lympho-sarcomatous growths by their spontaneous disappearance.

As bearing on this subject, I may mention that the late Mr. Greig Smith reported in the *Medico-Chirurgical Transactions* (1894, lxxvii. 139), three cases of solid abdominal tumours which spontaneously disappeared, and a discussion on this paper may be found in the *Proceedings of the Royal Medical and Chirurgical Society of London* (1894, 3. s. vi. 44).

